# Efficacy and safety analysis in metastatic cancer patients treated with multiple courses of repeat radiation therapy

**DOI:** 10.1016/j.ctro.2023.100687

**Published:** 2023-10-02

**Authors:** Maiwand Ahmadsei, Sebastian M. Christ, Tiuri E. Kroese, Anja Kühnis, Jonas Willmann, Panagiotis Balermpas, Nicolaus Andratschke, Stephanie Tanadini-Lang, Matthias Guckenberger

**Affiliations:** a Department of Radiation Oncology and Competence Center for Palliative Care, University Hospital and University of Zurich, Zurich, Switzerland; b Center for Proton Therapy, Paul Scherrer Institute, ETH Domain, Villigen, Switzerland

**Keywords:** Radiation therapy, Re-irradiation, Repeat radiotherapy, Toxicity, Local control

## Abstract

**Background and purpose:**

Due to advances in oncology, a growing proportion of patients is treated with repetitive courses of radiotherapy. The aim of this study is to analyze whether radiotherapy maintains its safety and efficacy profile in patients treated with multiple repeat courses of irradiation.

**Material and methods:**

All patients treated between 2011 and 2019 at our institution were screened for a minimum of five repeat irradiation courses, to analyze treatment characteristics, survival, safety and efficacy. The type of re-irradiation was classified according to ESTRO-EORTC consensus guidelines.

**Results:**

A total of n = 112 patients receiving n = 660 radiotherapy courses were included in this retrospective cohort study. The most frequent primary tumors were lung cancer in 41.9 % (n = 47) and malignant melanoma in 8.9 % (n = 10). The most frequent re-irradiation types were repeat irradiation and Type 2 re-irradiation in 309 (46.8 %) and 113 (17.1 %) cases, respectively. Median survival after the first course of radiotherapy was 3.6 (0.3–13.4) years. Response to radiotherapy was observed in 548 (83.0 %) cases and CTCAE toxicity grade ≥ 3 was observed in 21 (3.2 %) cases. An increasing number of RT courses (HR: 1.30, p=<0.0001), Type 1 re-irradiation (HR 3.50, p = 0.008) and KPS ≤ 80 % (HR: 2.02, p = 0.002) were associated with significantly worse treatment responses. Toxicity rates remained stable with increasing numbers of RT courses.

**Conclusion:**

Multiple courses of repeat radiotherapy maintain a favorable therapeutic ratio of high response combined with reasonable safety profile.

## Highlights

• An increasing number of patients are receiving multiple courses of radiotherapy, yet data on efficacy and safety is scarce.
• Therefore, this study aimed to analyze efficacy and safety in 112 patients receiving a minimum of five radiotherapy courses using the EORTC-ESTRO re-irradiation classification.
• Response to radiotherapy was observed in 548 (83.0 %) cases and CTCAE toxicity grade > 3 was observed in 21 (3.2 %) cases.
• An increasing number of RT courses, Type 1 re-irradiation and KPS ≤ 80 % were associated with significantly worse treatment responses, while toxicity rates remained stable with increasing numbers of RT courses.
• Multiple repeat radiotherapy with a minimum of five radiotherapy courses maintains a favorable therapeutic ratio of high response combined with reasonable safety profile.

## Introduction

Rapid advances in cancer diagnosis and treatment during the last decade have transformed cancer into a chronic disease [1]. This transformation was facilitated by improved systemic treatments, as well as continuous technological advances in surgery and radiation oncology [2], [3], [4].

As a result of improved survival, increasing numbers of patients are receiving multiple courses of radiotherapy (RT), with some centers reporting up to 25–30 % of their patients having a second or third course of conventional RT or stereotactic body radiotherapy (SBRT) [5], [6], [7]. Yet, despite a growing body of literature about patients treated with in-field re-irradiation, data for repeat organ RT is scarce and there exist even less data for safety and efficacy for repeat organ RT [8], [9]. Indications for multiple courses of curative radiotherapy entail secondary malignancies, loco-regional recurrence, oligometastatic disease recurrence as well as repetitive local symptoms with indication for palliative radiotherapy. The additional lack of a clear nomenclature of repetitive irradiation separating reirradiation from repeat or multiple RT has recently encouraged the medical community to propose a universal classification system in order to improve comparability and technical adjustments necessary for repeat or multiple RTs [6], [10]. The majority of existing literature studying multiple RTs is organ-specific (lung, prostate, brain or head and neck) and entails mainly two courses of RT [8], [11], [12], [13]. Literature about patients treated with more than two RT courses is primarily based on case reports [5], [14], [15]. This lack of data is the background of an uncertainty or concern, whether radiotherapy maintains its well documented safety and efficacy profile even when patients are treated with multiple and repetitive courses of radiation.

Therefore the aim of this retrospective single-center study was to analyze and report on the safety and efficacy of RT in cancer patients treated with multiple courses of repeat RT during their disease history.

## Material and methods

### Patient cohort

**Fig. 1:**
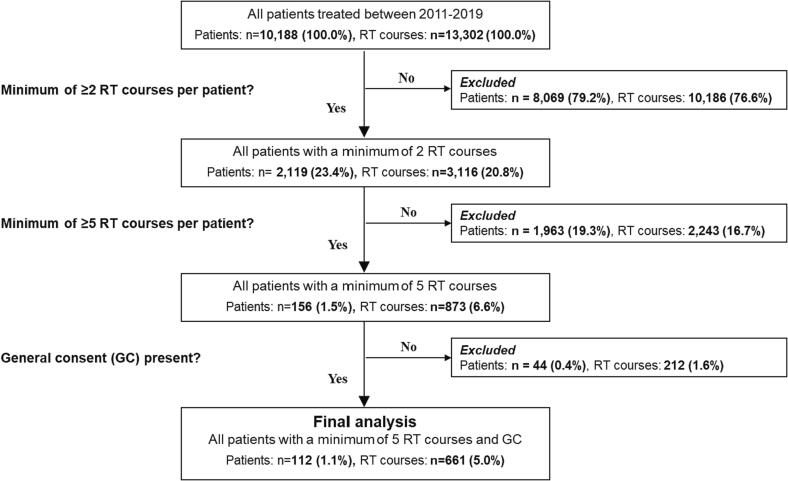
CONSORT diagram of inclusion criteria.

All patients treated between 2011 and 2019 at the University Hospital Zurich were included in this analysis and screened for treatment with multiple radiotherapy courses (n = 10.188). A course of radiotherapy (RT) was defined as a prescribed treatment to one anatomical site under the umbrella of one medical indication at one particular point in time in the patient history, as described previously [5]. After identification of all patients who received a minimum of two RT courses (n = 2.199), we selected all patients who were treated with a minimum of five RT courses (n = 121), as indicated in the CONSORT diagram (Fig. 1). We used the term multiple repeat RT (MRRT) to characterize a unique cohort of patients, who were treated with minimum five radiotherapy courses during their disease history. A minimum of five RT courses was chosen for inclusion into this study because of the lack of safety and efficacy data in the literature about such patients.

### Data collection

Patient, disease and treatment characteristics were extracted as previously described [5]. All Common Terminology Criteria for Adverse Events (CTCAE) grade ≥ 3 toxicities were documented in detail with date of occurrence and therapeutic management. Treatment response was evaluated using clinical information and imaging data from follow-up computer tomography (CT) and Fluorodeoxyglucose (18F)-Positron emission tomography–computed tomography (FDG-PET/CT). Successful treatment response for (*1*) palliative-analgesic RT was defined as: significant pain reduction (reduction of minimum 2 points on the visual analogue scale; subjective improvement as described by the patient in cases without available pain score data) reported by the patient within 8–12 weeks after treatment, for (*2*) extra-cranial local tumor control: morphologically-confirmed (CT or PET/CT) tumor size reduction and no local progression within 6 months after RT, and for *(3)* brain metastases: magnetic resonance imaging (MRI)-confirmed tumor size reduction and no local progression within 3 months after RT. After treatment patients were followed up 6 weeks and three months after RT to evaluate early toxicity. Afterwards, patients underwent imaging (MRI, CT or PET/CT) every three months during regular follow-up procedures. This project was approved by the Swiss Cantonal Ethics-Committee (BASEC# 2021–00104).

### Statistical analysis

Overall survival (OS) was calculated from the time of the first RT course and date of primary diagnosis to date of death or last follow-up. OS curves were estimated by using Kaplan-Meier method in R-Studio statistical software (Version 2022.07.1 + 554, R-package “survival”), as well as univariate and multivariate analysis using the Cox proportional hazard model. The associations between demographic and clinical characteristics were evaluated with two-sided nonparametric Wilcoxon rank sum test for continuous variables, and with two-sided Fisher’s exact test for categorical variables. Statistical significance was set at p < 0.05. Correction for multiple testing was conducted using Benjamini-Hochberg procedure.

## Results

### Patient characteristics

A total of 112 patients treated with a minimum of 5 RT courses between 2011 and 2019 at our institution were included in this study. The most common primary tumor were thoracic malignancies (n = 47; 41.9 %) - which included non-small cell lung cancer (NSCLC), small-cell lung cancer (SCLC) and mesothelioma - and malignant melanoma (n = 10; 8.9 %). The majority of patients (n = 83 patients; 74.0 %) presented metastatic disease at the time of first RT. Detailed patient characteristics are summarized in Table 1.

**Table 1:** Patient characteristics.

**Parameter**	**Data (n = 112 patients)**
Age at primary diagnosis in years, median (range)	56 (26–85)
Female gender, n (%)	51 (45.5)
Karnofsky performance status (KPS) at first RT course in %, median (range)	90 (60–100)
Charlson Comorbidity Index (CCI)at first RT course, median (range)	6 (0–12)
Median follow-up time in years (range)	3.7 (0.3–13.6)
Primary tumor histology, n (%)	
Lung^1^	47 (41.9)
NSCLC	40 (35.7)
SCLC	6 (5.3)
Mesothelioma	1 (0.9)
Malignant melanoma	10 (8.9)
Breast cancer	9 (8.0)
Soft tissue & bone	8 (7.1)
Colorectal	7 (6.3)
Head & neck	7 (6.3)
Other^1^	24 (21.4)
Metastatic disease at first RT course	83 (74.0)
Alive at time of analysis, n (%)	24 (21.4)

1 Includes prostate, urinary tract, endocrine, gynecologic, hematologic, esophageal and hepatocellular cancer entities as well as cancer of unknown origin.

### Treatment characteristics

All 112 patients were treated with a total of 660 RT courses. A detailed summary of treatment characteristics is shown in Table 2. The median total planning target volume (PTV) per course was 30.8 (0.1–6046.8) cm^3^, with great differences depending on RT site. The median PTV for brain metastases was 9.12 (range: 0.13–9.12) cm^3^, while bone metastases presented with a median PTV of 378.6 (range: 133.8–6046.8) cm^3^. The median cumulative PTV irradiated over all RT courses was 998.2 (range: 19.7–9958.1) cm^3^. The majority of RT courses were administered with a palliative intent (n = 513, 77.7 %). Whereas the first RT course was administered with a curative intent in roughly half of the patients, the proportion of curative intent fell to 11.6 % and 0 % at the 5th RT and 7-10th RT course, respectively. The median interval between primary diagnosis and first RT course was 8.2 months, for subsequent RT courses the median interval ranged between 1.7 and 6.8 months. The three most common treatment sites were bone (n = 265, 40.1 %), brain (n = 214, 32.4 %) and lung (n = 71, 10.1 %). The most frequent ESTRO-EORTC re-irradiation type [6] was repeat irradiation (n = 309 RT courses, 46.8 %), the second most frequent type of re-irradiation was Type 2 re-irradiation (re-irradiation with concerns of toxicity from cumulative doses without overlap of irradiated volumes) in 113 cases (17.1 %). The treatment indication was discussed in a multidisciplinary tumor board (MDT) in 402 cases (61.5 %) as shown in Supplementary Table 6.

**Table 2:** Treatment characteristics.

**Parameter**	**Data (n = 660 RT courses; n = 112 patients)**
**Number of radiotherapy fractions, median (range)**	6 (1–35)
**Dose per fraction in Gray, median (range)**	4 (1.8–20)
**Total dose in Gray, median (range)**	30 (3–70)
**Total RT volume in cm^3^, median (range)**	998.2 (19.7–9958.1)
**RT volume per course in cm^3^, median (range)**	30.8 (0.1–6046.8)
**Type of RT per course (n, %)**	
Conventional RT	380 (57.6)
SBRT	280 (42.4)
**Number of RT courses, median (range)**	5 (5–10)
**Number of RT courses per patient**	
5, n = patients (% of all patients)	60 (53.6)
6, n = patients (% of all patients)	25 (22.3)
7, n = patients (% of all patients)	14 (12.5)
8, n = patients (% of all patients)	8 (7.1)
9, n = patients (% of all patients)	3 (2.7)
10, n = patients (% of all patients)	2 (1.8)
**Treatment duration in days, median (range)**	14 (1–97)
**Interval (years) between first and last radiotherapy course, median (range)**	3 (0–8)
**Type of Re-irradiation per course (n, %)**	
No re-irradiation	112 (17.0)
Type 1 re-irradiation	70 (10.6)*
Type 2 re-irradiation	113 (17.1)
Repeat organ irradiation	56 (8.5)
Repeat irradiation	309 (46.8)
**Treatment before first RT course (n, %)**	
Surgery	60 (53.6)
Chemotherapy	62 (55.4)
Immunotherapy	8 (7.1)
Targeted therapy	10 (9.0)
**Systemic therapy within 30 days of any RT course (n, %)**	230 (34.8)
**Treatment intent**	
Curative, n (%)	147 (22.3)
Palliative, n (%)	513 (77.7)
**Treatment site**	
Bone, n (%)	265 (40.1)
Brain, n (%)	214 (32.4)
Lung, n (%)	71 (10.1)
Primary, n (%)	36 (5.4)
Lymph nodes, n (%)	29 (4.4)
Liver, n (%)	16 (2.4)
Soft tissue, n (%)	13 (2.0)
Adrenals, n (%)	9 (1.4)
Other, n (%)^1^	7 (1.1)

*Abbreviations*: RT = radiation therapy. *p = 0.0112 vs. no re-irradiation.

1 Includes mediastinum, kidneys, thyroid and pleura.

### Survival and treatment response

**Fig. 2:**
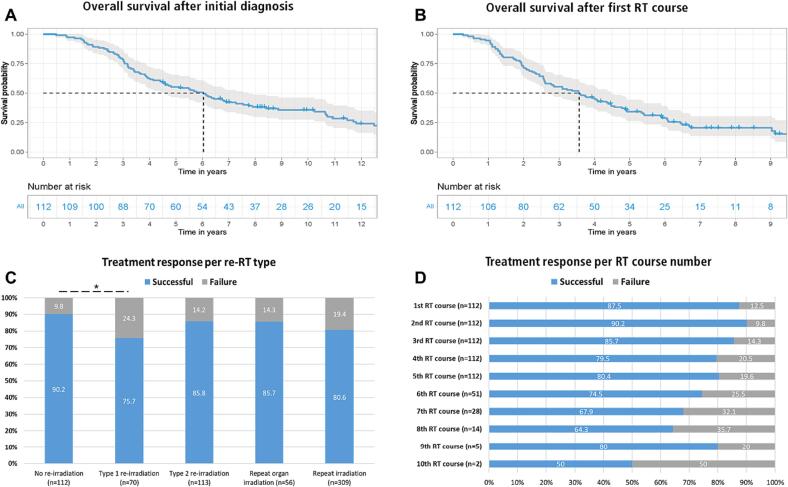
(A) overall survival after first RT course, (B) overall survival after initial diagnosis, (C) treatment response per re-irradiation type, (D) treatment response per RT course number.

At a median follow-up time of 3.7 (0.3–13.6) years, median OS after initial diagnosis and first RT course were 6.0 (0.5–26.0) years and 3.6 (0.3–13.4) years, respectively (Fig. 2A-B). The short-term RT course treatments response, as defined above, was scored as “successful” in 548 cases (83.0 %). The treatment response for the endpoints of “local tumor control” and “analgesia” was scored as successful in 367 RT courses (86.7 %) and 181 RT courses (76.3 %), respectively.

Furthermore, treatment response stratified over the ESTRO-EORTC re-irradiation classification [6] showed significantly lower rates of successful treatment response of Type 1 re-irradiation compared to non-re-irradiation (75.5 % vs. 90.2 %; p = 0.0112; Fig. 2C). In the univariate Cox regression analysis, total dose applied per RT course (HR: 0.97, p = 0.004) and concurrent chemotherapy at time of RT (HR: 0.61, p = 0.02) were associated with improved treatment response. In contrast, increasing number of RT courses (HR: 1.30, p=<0.0001), Type 1 re-irradiation (HR 3.51, p = 0.008) and Karnofsky performance status (KPS) ≤ 80 % (HR: 2.02, p = 0.002) were associated with significantly worse treatment response, a detailed summary of uni- and multivariate Cox regression analysis is shown in Table 4. A treatment response at the first RT course was observed in 87.5 % of the cases, while a response at the 5th was observed in 80.4 % cases (Fig. 2D). A detailed summary of survival and treatment response is illustrated in Table 3. The univariate Cox regression analysis did not detect any patient or treatment characteristic associated with survival after adjustment for multiple testing (Supplementary Table 5).

**Table 3:** Overview of survival, treatment response and univariate analysis.

**Survival at median follow-up of 3.7 (range: 0.3**–**13.6) years**	**Median OS**	**Median 5-year survival**
**From date of primary diagnosis, years (range); %**	6.0 (0.5–26.0)	55.4
**From 1st RT, years (range); %**	3.6 (0.3–13.4)	34.2
**Treatment response parameter, n (%)**	**Successful treatment response, n (%)**	
All RT courses (n = 660)	548 (83.0)	
Treatment response per RT indication		
Analgesia (n = 237)	181 (76.3)	
Local tumor control (n = 423)	367 (86.7)	
Treatment response per RT site		
Bone metastasis (n = 265)	205 (77.4)	
Brain metastasis (n = 214)	180 (84.1)	
Lung (n = 71)	64 (90.0)	
Primary tumor (n = 36)	31 (86.1)	
Other^1^ (n = 74)	66 (89.2)	
Treatment response per RT course number		
1th RT course (n = 112)	98 (87.5)	
2nd RT course (n = 112)	101 (90.2)	
3rd RT course (n = 112)	96 (85.7)	
4th RT course (n = 112)	89 (79.5)	
5th RT course (n = 112)	90 (80.4)	
6th RT course (n = 51)	38 (74.5)	
7th RT course (n = 28)	19 (67.9)	
8th RT course (n = 14)	9 (64.3)	
9th RT course (n = 5)	4 (80.0)	
10th RT course (n = 2)	1 (50.0)	
Treatment response per type of Re-RT		
No re-irradiation (n = 112)	101 (90.2)	
Type 1 re-irradiation (n = 70)	53 (75.7)	
Type 2 re-irradiation (n = 113)	97 (85.8)	
Repeat organ irradiation (n = 56)	48 (85.7)	
Repeat irradiation (n = 309)	249 (80.6)	

*Abbreviations*: OS = overall survival; RT = radiation therapy.

1 Includes adrenal, kidney, lymph node, soft tissue and thyroid metastases.

**Table 4:** Overview of all ≥ CTCAE grade 3 toxicity events according to CTCAE Version 5 and univariate and multivariate Cox regression analysis of clinical parameters associated with toxicity and treatment response.

**Toxicity per RT course**	**CTCAE Grade 3, n (%)**	**CTCAE Grade 4, n (%)**	**CTCAE Grade 5, n (%)**	**Total number of toxicities, n (%)**
All ≥ CTCAE grade 3 toxicity events	**20 (3.0)**	0	**1 (0.2)**	**21 (3.2)**
*Detailed classification*				
Radiodermatitis (acute)	**5 (0.8)**	0	0	**5 (0.8)**
Radionecrosis (late)	**1 (0.2)**	0	0	**1 (0.2)**
Pain (acute)	**6 (0.9)**	0	0	**6 (0.9)**
Esophagitis (acute)	**2 (0.3)**	0	0	**2 (0.3)**
Nausea (acute)	**2 (0.3)**	0	0	**2 (0.3)**
Radiation pneumonitis (late)	**1 (0.2)**	0	0	**1 (0.2)**
Cerebral edema (acute)	**1 (0.2)**	0	**1 (0.2)**	**2 (0.3)**
Urosepsis (acute)	**1 (0.2)**	0	0	**1 (0.2)**
Kidney injury (acute)	**1 (0.2)**	0	0	**1 (0.2)**

**Uni- and multivariate analysis**	**≥CTCAE grade 3 toxicity events HR (95 % CI)**			

*Variable*	UVA - HR (95 % CI)	P value	MVA - HR (95 % CI)	P value

Age at RT	1.01 (0.97–1)	0.91	1.0 (0.96–1.0)	0.92
KPS ≤ 80 %				
No	*Reference*	–	*Reference*	–
Yes	1.11 (0.5–2.7)	0.91	1.24 (0.4–3.3)	0.73
Charlson Comorbidity Index (CMI)				
0	*Reference*	–	*Reference*	–
1	0.82 (0.1–5.4)	0.91	0.83 (0.1–6.4)	0.86
2	0.31 (0.1–1.5)	0.72	0.24 (0.1–1.6)	0.14
3	0.63 (0.1–3.2)	0.91	0.58 (0.1–3.7)	0.56
>3	0.90 (0.2–4.2)	0.93	0.99 (0.2–1.3)	0.99
Total dose applied per course	1.01 (0.99–1.1)	0.81	1.03 (0.99–1.1)	0.19
Increasing RT course number	0.93 (0.7–1.2)	0.93	0.98 (0.74–1.3)	0.91
Lung cancer vs. all other	1.40 (0.96–2.0)	0.81	0.46 (0.2–1.2)	0.12
Concurrent chemotherapy with RT				
No	*Reference*	–	*Reference*	–
Yes	1.10 (0.4–3.0)	0.92	1.4 (0.2–1.2)	0.57
Type of RT				
No re-irradiation	*Reference*	–	*Reference*	–
Type 1 re-irradiation	1.10 (0.3–4.1)	0.91	1.80 (0.4–8.5)	0.52
Type 2 re-irradiation	0.81 (0.3–2.9)	0.91	1.21 (0.3–5.2)	0.81
Repeat organ irradiation	0.32 (0.1–2.9)	0.82	0.42 (0.1–4.0)	0.50
Repeat irradiation	0.31 (0.1–1.0)	0.72	0.31 (0.1–1.3)	0.12

**Uni- and multivariate analysis**	**Treatment response HR (95 % CI)**			

*Variable*	UVA – HR (95 % CI)	P value	MVA HR (95 % CI)	P value

Age at RT	0.99 (0.98–1)	0.42	0.98 (0.97–1.0)	0.106
KPS ≤ 80 %				
No	*Reference*	–	*Reference*	–
Yes	**2.02 (1.4**–**3.0)**	**0.002**	1.34 (0.9–2.0)	0.163
Charlson Comorbidity Index (CMI)				
0	*Reference*	–	*Reference*	–
1	0.32 (0.1–1.6)	0.21	0.31 (0.1–1.6)	0.16
2	1.10 (0.5–2.7)	0.92	1.10 (0.4–3.0)	0.92
3	2.01 (0.8–5.0)	0.24	1.92 (0.7–5.1)	0.21
>3	1.52 (0.6–3.9)	0.41	1.50 (0.5–4.2)	0.45
Total dose applied per course	**0.97 (0.95**–**0.99)**	**0.004**	**0.98 (0.96**–**1.0)**	**0.025**
Increasing RT course number	**1.30 (1.2**–**1.4)**	**0.000008**	**1.20 (1.1**–**1.3)**	**0.001**
Lung cancer vs. all other	1.42 (0.96–2.0)	0.22	**1.63 (1.1**–**2.4)**	**0.014**
Concurrent chemotherapy with RT				
No	*Reference*	–	*Reference*	–
Yes	**0.61 (0.4**–**0.9)**	**0.02**	**0.60 (0.4**–**0.9)**	**0.019**
Type of RT				
No re-irradiation	*Reference*	–	*Reference*	–
Type 1 re-irradiation	**3.51 (1.5**–**7.9)**	**0.008**	1.41 (0.6–3.5)	0.41
Type 2 re-irradiation	1.80 (0.8–4.2)	0.23	0.83 (0.3–2.0)	0.74
Repeat organ irradiation	1.62 (0.6–4.3)	0.41	0.90 (0.3–2.5)	0.82
Repeat irradiation	**2.71 (1.4**–**5.5)**	**0.014**	1.42 (0.6–3.0)	0.43

### Toxicity

Out of 660 administered RT courses, 21 RT courses (3.2 %) resulted in any ≥ CTCAE grade 3 toxicity events during follow-up. Majority of side effects were acute CTCAE grade 3 toxic events (20/21, 95.2 %). The most frequent acute CTCAE grade 3 toxicities were pain (n = 6, 0.9 %), radiodermatitis (n = 5, 0.8 %), esophagitis (n = 2, 0.3 %), nausea (n = 2, 0.3 %) and singular cases (n = 1, 0.2 %) of cerebral edema, kidney injury and urosepsis. Furthermore, one patient experienced acute fatal CTCAE grade 5 cerebral edema. Neither the number of radiotherapy courses nor the type of reirradiation were associated with the risk of grade 3 + toxicity. The univariate and multivariate Cox regression analysis did not detect any patient or treatment characteristics associated with ≥ CTCAE grade 3 toxicity events. Any acute or late grade ≥ 3 toxicity developed in 1.8 % and 2.1 % after radiotherapy courses 5 & 6 versus radiotherapy courses 7–––10, respectively. A detailed summary of toxicity events are illustrated in Table 4.

## Discussion

To investigate efficacy and safety of multiple repeat courses of radiotherapy, we identified a unique cohort of 112 patients treated with a minimum of five RT courses between 2011 and 2019. This analysis is to our best knowledge the first study which analyzed the efficacy and safety using the novel ESTRO-EORTC re-irradiation classification for cancer patients treated with multiple courses of radiotherapy. In the present analysis, MRRT with a median of five RT courses, maximum 10 in one patient, resulted in low levels of ≥ CTCAE grade 3 toxicity events, which did not increase with an increasing number of radiotherapy courses. While we observed that efficacy of repeat radiotherapy decreased over time, absolute efficacy of radiotherapy remained stable and should not discourage from choosing for treatment with repeat radiotherapy, if indicated based on existing guidelines.

Data on the frequency and tolerability of MRRT remains very limited and is primarily based on case reports [5], [16]. Our group reported in 2021, that the proportion of cancer patients treated with a minimum five courses of radiotherapy increased continuously from 0.9 % in 2011 to 6.5 % in 2019; Osorio et al. reported that in recent years 20–30 % of their patient present with the need for a second or third course of RT [5], [7].

Lung cancer was the most frequent primary diagnosis and accounted for a total of 41.9 % of patients. Despite continuous improvements in systemic therapies, the majority of patients develop drug resistance thereby creating the need for repeat RT [17], [18]. For oligometastatic NSCLC, Iyengar et al. [19] and Gomez et al. [20] reported an improved PFS and OS after consolidative SBRT compared to standard of care (SoC). Theelen et al. demonstrated improved OS and PFS after addition of SBRT to immunotherapy also in metastatic NSCLC [21]. Mueller at al., analyzing 44 patients with metastatic NSCLC of whom seven patients underwent three or more courses of RT, reported favorable a OS and a ≥ CTCAE grade 3 toxicity rate of 4.5 % [22].

In the present study, an increasing number of RT courses was not associated with ≥ CTCAE grade 3 toxicity events in uni- and multivariate Cox regression analysis, thereby indicating preserved safety with an increasing number of RT courses. Furthermore, the relatively large median cumulatively irradiated volume of 998.2 cm^3^ was also not associated with higher toxicity rates. This observation needs to be interpreted in the context of the type of reirradiation, which was “repeat reirradiation” according to the ESTRO-EORTC classification in almost 50 % of the cases. This lack of low-dose or high-dose overlap in the majority of patients most likely explains the favorable toxicity profile [23]. We are currently performing dose accumulation of all radiotherapy courses including dose conversion into biologically equivalent dose to further investigate this issue.

As toxicity is mainly associated with organ radiosensitivity and previously irradiated volume overlap, disease- and site-agnostic comparability between different multiple RT patient cohorts remains problematic [23]. In one of the very few reports on patients having received ≥ 5 RT courses, Singh et al. showed a case report that six courses of RT in a head-and-neck cancer patient resulted in a good quality of life, but xerostomia and nasogastric tube dependence [15]. This is of course not comparable to a repeat radiotherapy of different and/or less vulnerable organs [24]. A total of 70 RT courses (10.6 %) were classified as Type 1 re-irradiation (overlapping previously irradiated volumes) and 113 RT courses (17.1 %) were classified as Type 2 re-irradiation (with concerns of toxicity from the cumulative dose). Despite this relevant number of patients treated with type 1 and type 2 reirradiation, this was not associated with an increased risk of toxicity. This might be explained by extensive inter-disciplinary discussion between radiation oncologists and medical physicists before treatment, as well as the consequent use of EQD2 dose accumulation in the situation of reirradiation planning at our center, to respect cumulative organs-at-risk tolerance doses. Our experiences are in agreement with data in the literature, that increased rates of toxicity of reirradiation are mostly observed after high-dose type 1 reirradiation for locally recurrent primary tumors, whereas type 1 and type 2 reirradiation in palliative intent is usually well tolerated [6], [25], [26], [27]. Our study adds to this knowledge that toxicity remains low even in the situation of multiple courses of radiotherapy.

While a treatment response was scored in a total of 548 RT courses (83.0 %), the response rate for palliative-analgesic RT was 76.3 % - similar to results in existing literature [28]. The 3-month LC rate for brain metastases was 84.1 % and the 6-month LC rate for lung tumors was 90.0 %. Ogawa et al. analyzed 31 patients with in-field local tumor relapse of NSCLC or lung metastases, the authors achieved a 6-month LC of 80.0 % after the second course of RT (SBRT) [29]. Concerning re-irradiation of brain metastases, Fritz et al. evaluated the safety and efficacy of repeat stereotactic radiosurgery (SRS) for brain metastases. In this study of 45 patients harboring 197 brain metastases with 16 patients having received a minimum of three SRS courses, the local control after 12 months was 84.0 % [30]. In the present study we could observe a longitudinal change of treatment response. While treatment response after the first RT course was scored successful in 87.5 % of the cases (n = 98/112), the response rate decreased over time to 80.4 % (n = 90/112) after the 5th RT course and to to 67.9 % (19/28) after the 7th RT course. Despite this decline of response rate, 68 % is still a favorable response rate for patients having mostly limited therapeutic options at these late stages of their course of disease, having undergone a minimum of 5 RT courses and presumably several lines of systemic therapies. A theoretical biological explanation for this observation could be the increased proportion of RT-associated genomic events (small deletion burden) increasing with growing number of RT courses and thereby leading to a diminished tolerance as reported by Kocakavuk et al. [31]. Furthermore, the palliative intent, which constituted the absolute majority of RT indications and resulted in dose compromises may have contributed to this observation, too. In the present study, concurrent chemotherapy, and higher doses applied per RT course were associated with better treatment response in the uni- and multivariate Cox regression analysis. This confirms experiences from single-course radiotherapy and should also be considered as validation of the efficacy endpoints used in this study. Additional stratification over the novel ESTRO-EORTC re-irradiation classification [6] showed significantly lower response rates for Type 1 re-irradiation compared to no re-irradiation RT (p = 0.112).

The regular use of MDTs for cancer patients contributes to improved clinical outcome and more balanced treatment recommendations [32], [33], [34].

Christ et al. recently demonstrated that 56.0 % of primary oligometastatic cases were discussed in MDTs, of which more than 50 % received a local therapy [35]. Other studies have reported a usage of MDTs in only 39 % prior to RT for lung cancer patients [19], [20], [36], [37]. In the current study, 61.5 % of all RT courses were discussed at the internal MDT, for SBRT the proportion was even higher with 82.5 % of the cases (n = 231/660), thereby being higher than in the existing literature. Interestingly, the proportion of MDT-discussed cases decreases over time, while the first RT course was introduced at the MDT in 83.0 % of cases, the proportion at the fifth RT course and and sixth RT course shrinks to 52.7 % and 21.6 %, respectively. The majority of these cases can be explained by re-irradiation of bone- and brain metastases, where the patients are directly referred for palliative RT or are treated within the follow-up at our department.

Shortcomings of this study consist in its retrospective nature and the limited number of patients treated over a period of 10 years. We included highly selected patients who have received a minimum of five RT courses to assess the potential risk of cumulative toxicity. Additionally, due to the retrospective nature of this study, grade 2 toxicity could not be evaluated and was therefore intentionally not analyzed. Yet, for some patients grade 2 toxicity events might cause relevant symptoms, which might impact the treatment safety and efficacy. Furthermore, we evaluated short-term treatment responses to assess the short-term benefit MRRT. Yet, the strength of this study was the systematic approach to identify MRRT patients and analyze the efficacy and toxicity using the novel ESTRO-EORTC re-irradiation classification system. It is important to highlight that 67.0 % of the patients (n = 75/112) were treated within 3 years of the last study period, highlighting the increasing clinical relevance.

In conclusion, this study is the first to demonstrate that MRRT with a minimum of five RT courses maintains a favorable therapeutic ratio of high response combined with reasonable safety profile. Additional prospective data and more detailed dosimetric analyses will be required to further optimize treatment of this increasing patent population.

*The following parameters were defined as categorical variables: CCI, primary diagnosis: lung cancer, type of RT, concurrent chemotherapy at time of RT, KPS ≤ 80 %, while age, number of RT courses and total dose delivered per course were defined as a continuous variable. Correction for multiple testing was conducted using Benjamini-Hochberg procedure.*

## Funding

Maiwand Ahmadsei and Sebastian M. Christ received support through the “Young Talents in Clinical Research” Beginner’s Grant from the Swiss Academy of Medical Sciences (SAMW) and the Bangerter-Rhyner Foundation. This funding source had no role in the design of this study and will not have any role during its execution, analyses, interpretation of the data, or decision to submit results.

## Declaration of Competing Interest

The authors declare that they have no known competing financial interests or personal relationships that could have appeared to influence the work reported in this paper.

## References

[1] Sung H., Ferlay J., Siegel R.L., Laversanne M., Soerjomataram I., Jemal A. (2021). Global Cancer Statistics 2020: GLOBOCAN Estimates of Incidence and Mortality Worldwide for 36 Cancers in 185 Countries. CA Cancer J Clin.

[2] Dörr W., Gabryś D. (2018). The Principles and Practice of Re-irradiation in Clinical Oncology: An Overview. Clin Oncol.

[3] Qiu B, Aili A, Xue L, Jiang P, Wang J. Advances in Radiobiology of Stereotactic Ablative Radiotherapy. *Front Oncol*. 2020;10. Accessed March 18, 2023. https://www.frontiersin.org/articles/10.3389/fonc.2020.01165. 10.3389/fonc.2020.01165 PMC7426361 32850333

[4] Nieder C, Andratschke NH, Grosu AL. Increasing frequency of reirradiation studies in radiation oncology: systematic review of highly cited articles. PMC3623835 23593538

[5] Christ S.M., Ahmadsei M., Wilke L., Kühnis A., Pavic M., Tanadini-Lang S. (2021). Long-term cancer survivors treated with multiple courses of repeat radiation therapy. Radiat Oncol.

[6] Andratschke N., Willmann J., Appelt A.L., Alyamani N., Balermpas P., Baumert B.G. (2022). European Society for Radiotherapy and Oncology and European Organisation for Research and Treatment of Cancer consensus on re-irradiation: definition, reporting, and clinical decision making. Lancet Oncol.

[7] Vasquez Osorio E., Mayo C., Jackson A., Appelt A. (2023). Challenges of re-irradiation: A call to arms for physicists - and radiotherapy vendors. Radiother Oncol.

[8] Lee T.H., Kim D.Y., Wu H.G., Lee J.H., Kim H.J. (2021). Treatment outcomes of re-irradiation using stereotactic ablative radiotherapy to lung: a propensity score matching analysis. Radiat Oncol Lond Engl.

[9] Chargari C., Escande A., Dupuis P., Thariat J. (2022). Reirradiation: A complex situation. Cancer Radiother J Soc Francaise Radiother Oncol.

[10] Murray L., Thompson C., Pagett C., Lilley J., Al-Qaisieh B., Svensson S. (2023). Treatment plan optimisation for reirradiation. Radiother Oncol J Eur Soc Ther Radiol Oncol.

[11] Yan M., Lee M., Myrehaug S., Tseng C.-L., Detsky J., Chen H. (2023). Hypofractionated stereotactic radiosurgery (HSRS) as a salvage treatment for brain metastases failing prior stereotactic radiosurgery (SRS). J Neurooncol.

[12] Roesch J., Oertel M., Wegen S. (2022). Dose-escalated re-irradiation improves outcome in locally recurrent head and neck cancer - Results of a large multicenter analysis. Radiother Oncol J Eur Soc Ther Radiol Oncol.

[13] Miszczyk M, Kraszkiewicz M, Moll M, et al. Long-Term Outcomes of Stereotactic Body Radiotherapy (SBRT) for Intraprostatic Relapse after Definitive Radiotherapy for Prostate Cancer: Patterns of Failure and Association between Volume of Irradiation and Late Toxicity. . 2023;15(4):1180. 10.3390/cancers15041180. 10.3390/cancers15041180 PMC9954604 36831523

[14] Vucetic A., Ahmad B., Tang T. (2022). Long-term survival in a patient with extensive-stage small cell lung cancer treated with multiple courses of salvage stereotactic radiation after whole brain radiotherapy: A case report. Oncol Lett.

[15] Singh D., Goel A., Chaudhary P., Subramaniam B. (2022). Reirradiation and re-reirradiation in head-and-neck cancers: Learnings based on a case irradiated six times. J Cancer Res Ther.

[16] Volpe S., Jereczek-Fossa B.A., Zerini D., Rojas D.P., Fodor C., Vavassori A. (2019). Case series on multiple prostate re-irradiation for locally recurrent prostate cancer: something ventured, something gained. Neoplasma.

[17] Barton M.B., Allen S., Delaney G.P., Hudson H.M., Hao Z., Allison R.W. (2014). Patterns of Retreatment by Radiotherapy. Clin Oncol.

[18] Howlader N., Forjaz G., Mooradian M.J., Meza R., Kong C.Y., Cronin K.A. (2020). The Effect of Advances in Lung-Cancer Treatment on Population Mortality. N Engl J Med.

[19] Iyengar P., Wardak Z., Gerber D.E., Tumati V., Ahn C., Hughes R.S. (2018). Consolidative Radiotherapy for Limited Metastatic Non–Small-Cell Lung Cancer: A Phase 2 Randomized Clinical Trial. JAMA Oncol.

[20] Gomez D.R., Blumenschein G.R., Lee J.J., Hernandez M., Ye R., Camidge D.R. (2016). Local consolidative therapy versus maintenance therapy or observation for patients with oligometastatic non-small-cell lung cancer without progression after first-line systemic therapy: a multicentre, randomised, controlled, phase 2 study. Lancet Oncol.

[21] Theelen W.S.M.E., Chen D., Verma V., Hobbs B.P., Peulen H.M.U., Aerts J.G.J.V. (2021). Pembrolizumab with or without radiotherapy for metastatic non-small-cell lung cancer: a pooled analysis of two randomised trials. Lancet Respir Med.

[22] Muller D.A., Dutta S.W., Aliotta E., Sanders J.C., Wijesooriya K., Watkins W.T. (2021). Clinical Outcomes and Predictors of Lung Toxicity After Multiple Courses of Lung Stereotactic Body Radiotherapy for Early-Stage Non-Small Cell Lung Cancer. Clin Lung Cancer.

[23] Budach V, Thieme A. Re-Irradiation for Local Relapses or Second Primaries: When and how? In: Vermorken JB, Budach V, Leemans CR, Machiels JP, Nicolai P, O’Sullivan B, eds. *Critical Issues in Head and Neck Oncology*. Springer International Publishing; 2021:247-275. 10.1007/978-3-030-63234-2_17.

[24] Nieder C., Langendijk J.A., Guckenberger M., Grosu A.L. (2018). Second re-irradiation: a narrative review of the available clinical data. Acta Oncol Stockh Swed.

[25] Yang W.-C., Hsu F.-M., Chen Y.-H., Shih J.-Y., Yu C.-J., Lin Z.-Z. (2020). Clinical outcomes and toxicity predictors of thoracic re-irradiation for locoregionally recurrent lung cancer. Clin Transl Radiat Oncol.

[26] Chung SY, Koom WS, Keum KC, et al. Treatment Outcomes of Re-irradiation in Locoregionally Recurrent Rectal Cancer and Clinical Significance of Proper Patient Selection. *Front Oncol*. 2019;9. Accessed April 25, 2023. https://www.frontiersin.org/articles/10.3389/fonc.2019.00529. 10.3389/fonc.2019.00529 PMC6593136 31275858

[27] Embring A, Onjukka E, Mercke C, et al. Overlapping volumes in re-irradiation for head and neck cancer – an important factor for patient selection. *Radiat Oncol*. 2020;15(1):147. 10.1186/s13014-020-01587-3. 10.1186/s13014-020-01587-3 PMC7278185 32513217

[28] van der Velden J.M., van der Linden Y.M., Versteeg A.L., Verlaan J.-J., Sophie Gerlich A., Pielkenrood B.J. (2018). Evaluation of effectiveness of palliative radiotherapy for bone metastases: a prospective cohort study. J Radiat Oncol.

[29] Ogawa Y., Shibamoto Y., Hashizume C., Kondo T., Iwata H., Tomita N. (2018). Repeat stereotactic body radiotherapy (SBRT) for local recurrence of non-small cell lung cancer and lung metastasis after first SBRT. Radiat Oncol.

[30] Fritz C, Borsky K, Stark LS, et al. Repeated Courses of Radiosurgery for New Brain Metastases to Defer Whole Brain Radiotherapy: Feasibility and Outcome With Validation of the New Prognostic Metric Brain Metastasis Velocity. *Front* Oncol. 2018;8. Accessed March 18, 2023. https://www.frontiersin.org/articles/10.3389/fonc.2018.00551. 10.3389/fonc.2018.00551 PMC6262082 30524969

[31] Kocakavuk E, Anderson KJ, Varn FS, et al. Radiotherapy is associated with a deletion signature that contributes to poor outcomes in cancer patients. *Nat Genet*. 2021;53(7):1088-1096. 10.1038/s41588-021-00874-3. 10.1038/s41588-021-00874-3 PMC8483261 34045764

[32] Brauer D.G., Strand M.S., Sanford D.E., Kushnir V.M., Lim K.-H., Mullady D.K. (2017). Utility of a multidisciplinary tumor board in the management of pancreatic and upper gastrointestinal diseases: an observational study. HPB.

[33] Tamburini N, Maniscalco P, Mazzara S, et al. Multidisciplinary management improves survival at 1 year after surgical treatment for non-small-cell lung cancer: a propensity score-matched study†. . 10.1093/ejcts/ezx464 29293943

[34] Ioannidis A, Konstantinidis M, Apostolakis S, Koutserimpas C, Machairas N, Konstantinidis KM. Impact of multidisciplinary tumor boards on patients with rectal cancer. *Mol Clin Oncol*. 2018;9(2):135-137. 10.3892/mco.2018.1658*Clin Transl Radiat Oncol*. 2023;38:123-129. 10.1016/j.ctro.2022.11.008. 10.3892/mco.2018.1658 PMC6083403 30101009

[35] Christ S.M., Heesen P., Muehlematter U.J., Pohl K., William Thiel G., Willmann J. (2023). Recognition of and treatment recommendations for oligometastatic disease in multidisciplinary tumor boards. Clin Transl Radiat Oncol.

[36] Gomez D.R., Tang C., Zhang J., Blumenschein G.R., Hernandez M., Lee J.J. (2019). Local Consolidative Therapy Vs. Maintenance Therapy or Observation for Patients With Oligometastatic Non–Small-Cell Lung Cancer: Long-Term Results of a Multi-Institutional, Phase II, Randomized Study. J Clin Oncol.

[37] De Ruysscher D., Wanders R., van Baardwijk A., Dingemans A.-M., Reymen B., Houben R. (2012). Radical treatment of non-small-cell lung cancer patients with synchronous oligometastases: long-term results of a prospective phase II trial (Nct01282450). J Thorac Oncol off Publ Int Assoc Study Lung Cancer.

