# Supplementary material for: Efficacy and safety analysis in metastatic cancer patients treated with multiple courses of repeat radiation therapy
Source: Clin Transl Radiat Oncol. 2023 Oct 2;43:100687. doi: 10.1016/j.ctro.2023.100687 (PMC10589769; doi:10.1016/j.ctro.2023.100687)
Supplement: Supplementary data 1 [file mmc1.docx]

**Supplementary material:**

**Table 5:** Univariate and multivariate Cox regression analysis of clinical parameters associated with overall survival

| **Endpoint** | **Overall survival HR (95% CI)** | | | |
| --- | --- | --- | --- | --- |
| *Variable* | UVA | P value | MVA | P value |
| Age | 1 (0.98-1.02) | 0.98 | 1.0 (0.98-1.0) | 0.868 |
| Sex   - Female - Male | *Reference*  1.1 (0.7-1.7) | -  0.88 | -  1.09 (0.7-1.7) | -  0.715 |
| Charlson Comorbidity Index (CCI)  o 0  o 1  o 2  o 3  o >3 | Reference  0.5 (0.2-1.8)  0.7 (0.3-1.6)  0.8 (0.4-2.0)  1.1 (0.5-2.4) | -  0.64  0.67  0.88  0.89 | -  0.49 (0.14-1.7)  0.65 (0.28-1.5)  0.93 (0.38-2.3)  1.17 (0.47-2.9) | -  0.254  0.317  0.867  0.737 |
| Lung cancer vs. all other | 1.3 (0.8-1.9) | 0.64 | 1.13 (0.72-1.8) | 0.595 |
| Metastatic disease at first RT course?  No  Yes | Reference  1.3 (0.9-2.1) | -  0.64 | -  1.36 (0.83-2.2) | -  0.226 |
| Concurrent systemic therapy at first RT course?  No  Yes | Reference  1.5 (0.9-2.5) | -  0.36 | -  **1.82 (1.01-3.3)** | -  **0.048** |
| KPS ≤80%  No  Yes | Reference  1.2 (0.8-1.9) | -  0.64 | -  1.24 (0.77-2.0) | -  0.372 |

*The following parameters were defined as categorical variables: sex, CCI, primary diagnosis: lung cancer, metastatic disease at time of first RT, concurrent chemotherapy at time of first RT, KPS ≤80%, while age was defined as a continuous variable. Correction for multiple testing was conducted using Benjamini-Hochberg procedure.*

**Table 6:** *Overview of RT indications discussed at MDT*

| **RT indication discussed at MDT (n, %)**   - All RT courses (n=660) - Only SBRT (n=280) - 1^th^ RT course (n=112) - 2^nd^ RT course (n=112) - 3^rd^ RT course (n=112) - 4^th^ RT course (n=112) - 5^th^ RT course (n=112) - 6^th^ RT course (n=51) - 7^th^ RT course (n=28) - 8^th^ RT course (n=14) - 9^th^ RT course (n=5) - 10^th^ RT course (n=2) | **n, %**  402 (61.5)  231 (82.5)  93 (83.0)  89 (79.5)  79 (70.5)  54 (48.2)  59 (52.7)  11 (21.6)  10 (35.7)  4 (28.6)  2 (40.0)  1 (50.0) |
| --- | --- |
